# Isolation of plant growth-promoting rhizobacteria from wild-simulated ginseng and evaluation of soil health following its application in the field

**DOI:** 10.3389/fmicb.2025.1682016

**Published:** 2025-11-25

**Authors:** Seok Hui Lee, Yeong-Bae Yun, Dae Sol Kim, Myeongbin Park, Yurry Um, Jun Won Kang

**Affiliations:** 1 Department of Forestry, School of Forest Sciences and Landscape Architecture, Kyungpook National University, Daegu, Republic of Korea; 2 Forest Medicinal Resources Research Center, National Institute of Forest Science, Yeongju, Republic of Korea

**Keywords:** *Paenibacillus terrae*, *Paraburkholderia madseniana*, *Paraburkholderia terricola*, plant growth-promoting rhizobacteria, *Pseudomonas frederiksbergensis*, soil stability

## Abstract

Wild-simulated ginseng must be cultivated at natural forest sites without artificial structures, chemical fertilizers, or pesticides to qualify for certification. However, its extended cultivation period makes stable production challenging, necessitating effective strategies to enhance early growth and yield. In this study, we evaluated the ability of five bacterial strains isolated from the rhizosphere of wild-simulated ginseng to promote initial growth and development. The strains exhibited diverse functional traits, including indole-3-acetic acid (IAA) production, phosphate solubilization, siderophore production, and enzymatic activities such as protease and cellulase. Antifungal activity, however, was primarily observed in strains 79 and 81. We inoculated field-grown ginseng plants with each strain at biweekly intervals for a total of seven applications. Inoculation with strain 75 (*Pseudomonas frederiksbergensis*) significantly increased shoot dry weight by 48.9% and root biomass by 37.0% relative to uninoculated controls (*p* < 0.05). Strain 81 (*Paenibacillus terrae*) promoted stem elongation, whereas strain 89 (*Paraburkholderia madseniana*) reduced leaf size. Soil analysis showed that strain 75 and 77 plots maintained higher organic matter, phosphorus, calcium, and cation exchange capacity, whereas strains 79, 81, and 89 had lower values. Metagenomic analysis revealed a marked enrichment of the order Pseudomonadales and the maintenance or enhancement of bacterial alpha diversity (Chao1 and PD indices), suggesting a stable and resilient microbial ecosystem. Functional profiling revealed enhancements in nitrogen fixation and nutrient cycling pathways. We determined statistical significance using a *t*-test and one-way ANOVA with Duncan’s multiple range test (*p* < 0.05). In contrast, strains 79 (*Paraburkholderia terricola*), 81 (*Paenibacillus terrae*), and 89 (*Paraburkholderia madseniana*) did not elicit significant growth responses. Our findings indicate that *P. frederiksbergensis* (strain 75) is a promising plant growth-promoting rhizobacterium for wild-simulated ginseng, offering a biologically based approach for improving early-stage development under forest cultivation conditions.

## 1 Introduction

Wild-simulated ginseng (*Panax ginseng*) is a perennial herbaceous plant belonging to the family Araliaceae. The main benefits of wild-simulated ginseng include activation of the immune response (Jung et al., 2019), antioxidant effects (Kwon et al., 2012), enhancement of liver function (Lee et al., 2008), anticancer effects (Jin et al., 2004), and antiobesity effects (Jeon et al., 2012). In addition, recent studies have suggested potential cosmetic applications (Kim M. W. et al., 2018). Ginsenosides, the main pharmacologically active compounds in wild-simulated ginseng, are rarely found in other plants. Polysaccharides and phenolic compounds also contribute to anticancer and antioxidant effects (Nam et al., 2015). Although cultivated ginseng also contains ginsenosides, wild ginseng has been reported to contain higher concentrations and to exhibit stronger anticancer and antioxidant activities. However, wild ginseng is low-yielding and expensive. Therefore, the demand for wild-simulated ginseng has been steadily increasing, and harvesting technology has been developed to enhance its medicinal properties (Ahn et al., 2007; Jeong et al., 2010).

Korean wild-simulated ginseng is internationally recognized for its superior quality and is traded at high prices in global markets. However, because of climate change, certain regions in Korea are facing difficulties in cultivating wild-simulated ginseng. Consequently, its production is declining. If climate change continues, the production of wild-simulated ginseng is expected to become increasingly unstable, which may further drive up its market price.

For the certification and sale of wild-simulated ginseng in the Republic of Korea, artificial shading, chemical pesticides, and fertilizers must not be used (National Law Information Center, 2023a,b). Wild-simulated ginseng increases in length and thickness for at least 5 years. Ginsenoside content also increases as the plant ages (Jeong et al., 2019). However, as the cultivation period extends, the risk of disease also increases. Previous research has revealed that when root rot pathogens (*Cylindrocarpon destructans* or *Fusarium* spp.) are present in the soil, 80% of 1- or 2-year-old ginseng plants become infected (Lee et al., 2014). On the other hand, 3- or 4-year-old ginseng plants exhibit higher resistance to root rot disease, although lesions may still appear on their root surface (Lee et al., 2014). Thus, plants in the early stages of growth are vulnerable to biotic and abiotic stresses. Therefore, research on strategies to promote early-stage growth and prevent disease is essential for increasing the yield of wild-simulated ginseng.

Wild-simulated ginseng is cultivated primarily for its roots, and increased root biomass is directly associated with higher yields. Nevertheless, recent research interest has shifted from biomass alone to root quality, particularly the accumulation of pharmacologically active compounds such as ginsenosides (Kim et al., 2021). Because of its inherently slow growth rate, wild-simulated ginseng is highly sensitive to soil conditions, including pH and organic matter content, which highlights the necessity of continuous soil management to ensure stable growth and development (Lee et al., 2025).

Recently, the main research directions have focused on identifying optimal cultivation regions by assessing environmental factors (e.g., tree species, surrounding vegetation, topography, altitude, and soil properties) or by using geographic information systems (GIS) (Kim J. Y. et al., 2018; Kim et al., 2019a). However, the use of GIS has not yet been validated through field cultivation tests. Moreover, predicting the damage caused by climate change is challenging because of the long cultivation period (Gyu et al., 2022). Proactive measures are necessary to ensure a stable yield of wild-simulated ginseng. In addition, biological control methods should be considered. A previous study reported that the growth of wild-simulated ginseng correlates with rhizosphere bacterial communities (Kim et al., 2019b).

Rhizosphere bacteria support plant growth by decomposing organic matter (OM), secreting hormones, facilitating nutrient uptake, and exhibiting antipathogenic activities (Baldrian, 2017). These bacteria are therefore known as plant growth-promoting rhizobacteria (PGPR). PGPR with high agricultural applicability are referred to as biocontrol agents (BCAs) (Jha and Saraf, 2015; Lahlali et al., 2022). Although the use of PGPR for enhancing the yield of cultivated ginseng has been actively studied (Dong et al., 2019; Kang et al., 2021), their application in wild-simulated ginseng remains underexplored. The forest environment differs from farmland, making it more difficult to manage plant growth (Zajícová and Chuman, 2019). Therefore, the isolation and application of PGPR from forest soil should be further investigated.

In this study, we isolated various rhizosphere bacteria and evaluated their antagonistic activity against *Fusarium* spp. (causing fusarium wilt) and *Colletotrichum* spp. (causing anthracnose), in addition to assessing their hormone production abilities and enzyme activities (Huo et al., 2021; Kim et al., 2024). Moreover, we selected the most effective strains and inoculated them into wild-simulated ginseng. In particular, the bacteria were assessed for their effects on plant initial growth and development and soil health.

## 2 Materials and methods

### 2.1 Isolation and identification

Root samples for bacterial isolation were collected from 7- and 13-year-old ginseng plants harvested in Pyeongchang, Muju, and Yeongju, Republic of Korea. After washing the root samples, their surfaces were sterilized by shaking them in 70% ethanol for 15 s and then in 0.5% NaClO for 3 min. After a second wash, the roots were sliced and placed on the following media: tryptic soy agar (TSA, MBcell, Seoul, Republic of Korea) (L^−1^: Tryptone, 17 g; Soytone, 3 g; Dextrose, 2.5 g; NaCl, 5 g; K₂HPO₄, 2.5 g; and micro agar, 15 g), 1/10 TSA, potato dextrose agar (PDA, MBcell, Seoul, Republic of Korea) (L^−1^: Infusion from potatoes, 7 g; Dextrose, 20 g; and micro agar, 15 g), and nutrient agar (NA, MBcell, Seoul, Republic of Korea) (L^−1^: Peptone, 5 g; beef extract, 3 g; and micro agar, 15 g). The strains selected for field trials were identified by 16S rRNA sequencing. PCR amplification was conducted by Macrogen Inc. (Seoul, Republic of Korea) using the primer set 27F (5′-AGAGTTTGATCMTGGCTCAG-3′) and 1492R (5′-TACGGYTACCTTGTTACGACTT-3′) (Frank et al., 2008). The resulting sequences were analyzed using the BLASTn tool against the National Center for Biotechnology Information (NCBI) database (Bethesda, MD, United States) (Sayers et al., 2023). A phylogenetic tree was constructed using MEGA v.11 with the maximum composite likelihood substitution model and 1,000 bootstrap replications (Tamura et al., 2021).

### 2.2 Antagonistic activity test

Effective strains were selected by assessing antifungal activity and hormone and enzyme production abilities. The pathogenic fungi used in the antifungal activity tests were *Colletotrichum gloeosporioides* (KACC No. 40003), *Fusarium solani* (KACC No. 40384), and *Fusarium oxysporum* (KACC No. 48505). Each pathogenic fungus was cultured on PDA for 2 weeks. The selected strains were cultured in tryptic soy broth (TSB, MBcell, Seoul, Republic of Korea) for 2 days at 27 °C. Subsequently, 4-mm agar plugs were cut from the fungal cultures and placed onto new PDA plates. In total, 3 μL of the test strain was inoculated onto sterile paper disks positioned 30 mm from the agar plugs and incubated at 27 °C. After measuring the distance between the test strain and the fungal mycelia, the antagonistic effects were assessed according to the following Equation 1 (Lee et al., 2023):


(1) $$\text{Inhibition rate}\;(\%)=\frac{\mathrm{Rt}}{\mathrm{Rc}}\times 100$$

where *Rc* represents the average growth radius of the mycelia on the control plate and *Rt* represents the distance between the mycelia and bacteria on the treated plates.

### 2.3 Enzyme and hormone production assays

Various enzyme and hormone production assays were performed to evaluate the initial growth-promoting effects and determine the antifungal mechanisms. These assays included the assessment of indole-3-acetic acid (IAA) production, phosphate solubilization, nitrogen fixation, siderophore production, chitinase activity, protease activity, and cellulase activity. Phosphate solubilization was assessed using National Botanical Research Institute’s phosphate growth medium (NBRIP, L^−1^: glucose, 10 g; Ca_3_(PO_4_)_2_, 5 g; MgCl_2_∙6H_2_O, 5 g; MgSO_4_, 0.14 g; KCl, 0.2 g; (NH_4_)_2_SO_4_, 0.1 g; and micro agar, 15 g). Protease production was evaluated on PDA supplemented with 2% skim milk (Nautiyal, 1999; Ahmad et al., 2014). Cellulase production was assessed on PDA supplemented with 1% carboxymethyl cellulose (CMC) and 0.01% trypan blue (Gohel et al., 2014). Nitrogen fixation was tested using Jensen’s medium (L^−1^: sucrose, 10 g; K_2_HPO_4_, 1 g; MgSO_4_, 0.5 g; NaCl, 0.5 g; FeSO_4_, 0.1 g; Na_2_MoO_4_, 0.005 g; CaCO_3_, 2 g; bromothymol blue, 0.25 g; and micro agar, 15 g) (Sulistiyani and Meliah, 2017). Siderophore production was assessed using the Chrome Azurol S (CAS) assay (Louden et al., 2011). Chitinase production was assessed on PDA containing 1% colloidal chitin, which was prepared according to a previously described method (Lee et al., 2023).

IAA production was evaluated using the Salkowski reaction after culturing each strain in TSB supplemented with 1% L-tryptophan for 2 days (Glickmann and Dessaux, 1995). total of 1.5 mL of the cultured broth was centrifuged for 10 min at 7300 × g at 4 °C, and the supernatant was mixed with 3 mL of Salkowski reagent (35% HClO_4_, 98 mL;0.5 M FeCl_3_, 2 mL) and incubated for 30 min at 25 °C in the dark. The concentration of IAA was quantified at 530 nm using a UV–Vis spectrophotometer (Ubi-490, MicroDigital Co., Ltd., Seongnam, Republic of Korea) against a standard curve. For phosphate solubilization, nitrogen fixation, siderophore production, and enzymatic activities of chitinase, protease, and cellulase, 3 sterile filter paper disks were placed on the surface of the respective media, and 3 μL of each overnight culture was inoculated onto the disks. Nitrogen fixation was considered positive when a blue-colored zone appeared, siderophore and cellulase activities were indicated by halo formation, and phosphate solubilization, chitinase, and protease activities were confirmed by the appearance of clear zones around the disks.

### 2.4 Field tests

Finally, five prospective strains were selected based on their functional traits, inoculated into wild-simulated ginseng in the field, and subsequently monitored. Inoculations were performed at biweekly intervals for a total of seven applications. After culturing 1 mL of each strain in 1 L of TSB for 3 days at room temperature, the culture broth was centrifuged at 1,666 × g for 10 min. Subsequently, the supernatant was removed. The cell pellets were then resuspended in sterile tap water and stored at 5 °C. Following this, 50 mL of the bacterial suspension was mixed with 5 L of tap water and sprayed onto the aboveground parts of individual plants, while 300 mL was drenched into the rhizosphere per plant. Control plants were treated with the same volumes of sterile tap water without bacterial inoculum.

From May 21 to August 7, a total of seven inoculations were performed. Each treatment included five biological replicates (*n* = 5). Among the 14 initial samples, several outliers caused by diseased or dead plants were excluded, and only the five tallest wild-simulated ginseng specimens were selected for measurement. Growth assessment parameters included stem length, stem diameter, leaf number, leaf area, rhizome head length, root diameter, root length, root hair number, fresh weight, and dry weight. Statistical analyses were conducted in R (v.4.2.1) using a *t*-test and one-way ANOVA with Duncan’s multiple range test (R Core Team, 2013).

### 2.5 Soil property analysis

Changes in the soil environment following strain inoculation were assessed using chemical and metagenomic analyses of soil samples. After removing the organic layer, soil samples were collected at a depth of 0–10 cm. Soil pH and electrical conductivity (EC) were determined using a pH meter and an EC meter, respectively, after mixing the soil with distilled water in a 1:5 ratio and agitating for 30 min. The organic matter (OM) content was assessed via the Walkley–Black method (Mylavarapu et al., 2014), while the total nitrogen (TN) content was measured using the Kjeldahl distillation method after treating 1 g of soil with 5 mL of concentrated sulfuric acid and processing it in a block digester (Bradstreet, 1954). The available phosphate (Avail. P) content was quantified by absorbance using 1-amino-2-naphthol-sulfanic acid via the Lancaster leaching method (Cox, 2001). The exchangeable cation content was determined through Inductively Coupled Plasma Optical Emission Spectrometry (ICP-OES) after leaching the soil with 1 N-ammonium acetate (NH_4_OAc), and cation exchange capacity (CEC) was measured by the Kjeldahl distillation of substituted NH_4_^+^ in the soil after leaching with 1 N-NH_4_OAc (Kim et al., 2025).

DNA extraction was performed using the DNeasy® PowerSoil® Pro Kit (Qiagen, Hilden, Germany), and DNA quality was assessed using a Nanodrop spectrophotometer (ND-LITE-PR, Thermo Fisher Scientific, Waltham, MA, United States). DNA sequencing was performed at the Kyungpook National University NGS Center (Daegu, Republic of Korea). Bacterial 16S rRNA V4 region amplicon sequencing was performed using the MiSeq system (Illumina, CA, United States), and fungal ITS2 region amplicon sequencing was performed using the HiSeq 3000 system (Illumina, CA, United States).

Raw DNA data were processed for filtering, phylogenetic tree construction, taxonomic classification, and normalization using Quantitative Insights Into Microbial Ecology 2 (QIIME2) and Divisive Amplicon Denoising Algorithm 2 (DADA2) (Callahan et al., 2016; Bolyen et al., 2019). Taxonomic classification was performed using the SILVA database (v.138) for bacteria and the UNITE database (v.8.3) for fungi (Bokulich et al., 2018; Kõljalg et al., 2020). Phylogenetic trees of bacterial sequences were constructed using SATé-enabled phylogenetic placement (SEPP), and fungal sequences were aligned using MAFFT v7 (Mirarab et al., 2012; Katoh and Standley, 2013). Alpha and beta diversity analyses were performed using the microeco package (v.1.11.0) in R (v.4.2.1) (Lozupone et al., 2011; Willis, 2019; Liu et al., 2021). The correlation between beta diversity and soil chemical properties was analyzed using distance-based redundancy analysis (dbRDA) and the Mantel test. Microbial functional traits were predicted using the FUNGuild database (Nguyen et al., 2016).

### 2.6 Network analysis of soil microbial community

Network analysis was performed to assess key taxa and their interactions using the Molecular Ecological Network Analysis Pipeline (MENAp) based on random matrix theory (RMT) (Deng et al., 2012). Only taxa present in at least 50% of soil samples were retained for network construction. The similarity matrix was calculated using Pearson correlation coefficients, and modules were detected via greedy modularity optimization (Newman, 2004). Modules with fewer than five nodes were excluded from module eigengene analysis. Nodes were classified into the following four topological roles based on their within-module connectivity (Zi) and among-module connectivity (Pi) values: (1) peripherals (Zi < 2.5, Pi < 0.62), (2) module hubs (Zi > 2.5, Pi < 0.62), (3) connectors (Zi < 2.5, Pi > 0.62), and (4) network hubs (Zi > 2.5, Pi > 0.62) (Guimera and Amaral, 2005).

## 3 Results

### 3.1 Bacterial isolation and identification

A total of 210 bacterial strains were isolated from the root tissue of wild-simulated ginseng, 80 strains were recovered from TSA, 13 on 1/10 TSA, 46 on PDA, and 51 on NA. Based on subsequent preliminary tests, including antifungal activity and IAA production assays, 20 strains were selected for the main experiments (Supplementary Table S1). The five final selected strains were identified using the NCBI database. Strains 75 and 77 were identified as *Pseudomonas frederiksbergensis* (NR 177277.1), strain 79 as *Paraburkholderia terricola* (NR 029044.1), strain 81 as *Paenibacillus terrae* (NR 025170.1), and strain 89 as *Paraburkholderia madseniana* (NR 180709.1) (Supplementary Figure S1).

### 3.2 *In vitro* tests

All five tested strains (79, 81, 85, 200, and 210) exhibited antifungal activity against *Fusarium* sp. and *Colletotrichum* sp. Moreover, enzyme production assays revealed that four strains (81, 85, 200, and 210) exhibited cellulase and protease activity (Supplementary Table S1; Supplementary Figure S2). However, strain 79 did not exhibit cellulase or protease activity associated with antifungal effects. Only strain 73 exhibited chitinase production ability, and only strain 79 exhibited nitrogen fixation ability. Four strains (75, 79, 89, and 210) were able to produce IAA. Among them, strain 89 showed the highest IAA production (19.20 ± 4.21 ppm). Additionally, 10 strains exhibited phosphate solubilization activity, and five strains exhibited siderophore production ability (Supplementary Table S1). Finally, five strains (75, 77, 79, 81, and 89) were selected for field testing.

### 3.3 Field inoculation test

**Figure 1:**
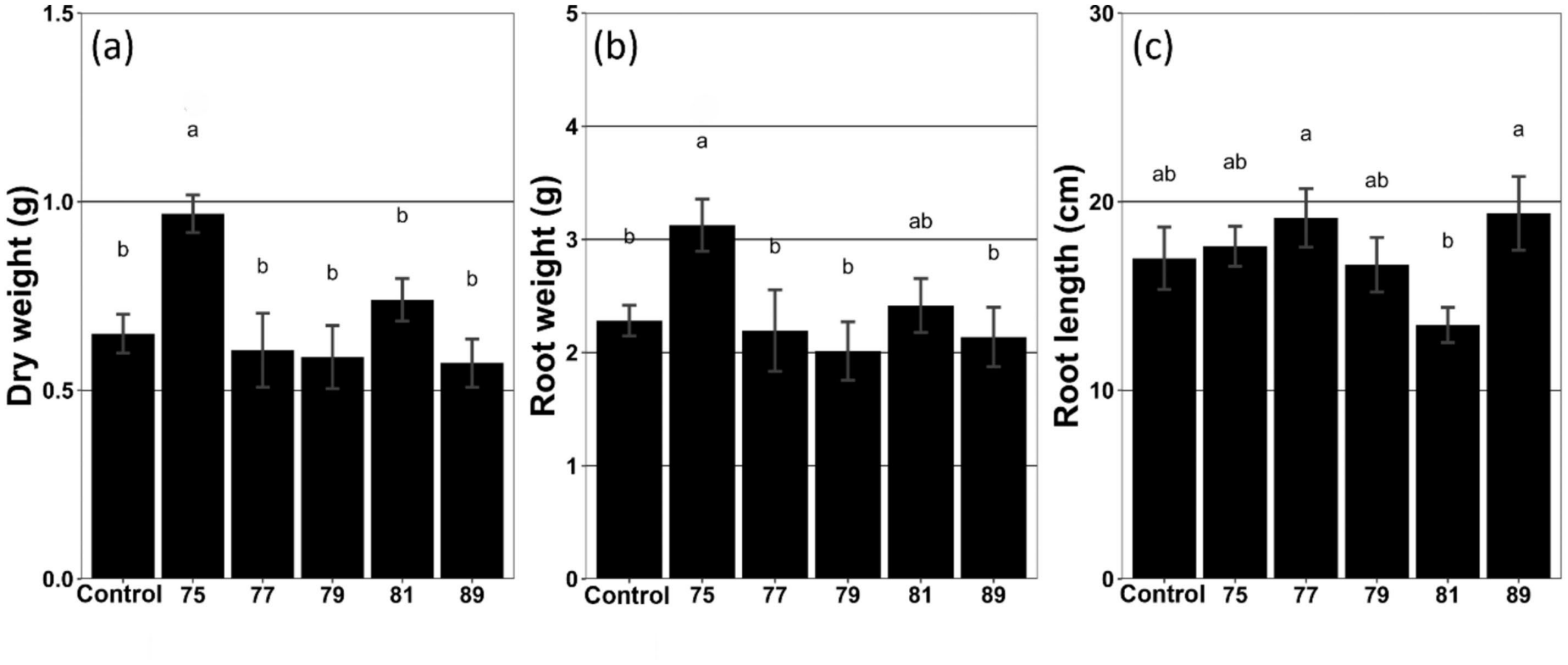
Growth characteristics of wild-simulated ginseng in response to inoculation with different bacterial strains (*n* = 5). **(a)** Dry weight (g), **(b)** root weight (g), and **(c)** root length (cm) are presented. Different letters above the bars indicate significant differences based on Duncan’s multiple range test following ANOVA (*p* < 0.05).

As a result of the inoculation tests and subsequent statistical analyses (ANOVA and t-tests), plots treated with strain 75 showed a significant increase in root weight (37.0%), fresh weight (48.5%), and dry weight (48.9%) (Figure 1; Supplementary Figure S3). Moreover, the mean leaf size also increased by 21.1% compared with the control (Supplementary Figure S3). The plot treated with strain 81 also exhibited increased growth; however, only the increase in stem length was statistically significant (21.1%) (Supplementary Figure S3). In contrast, the plot treated with strain 89 exhibited decreased mean leaf size (Supplementary Figure S3).

### 3.4 Soil metagenomic analysis

**Figure 2:**
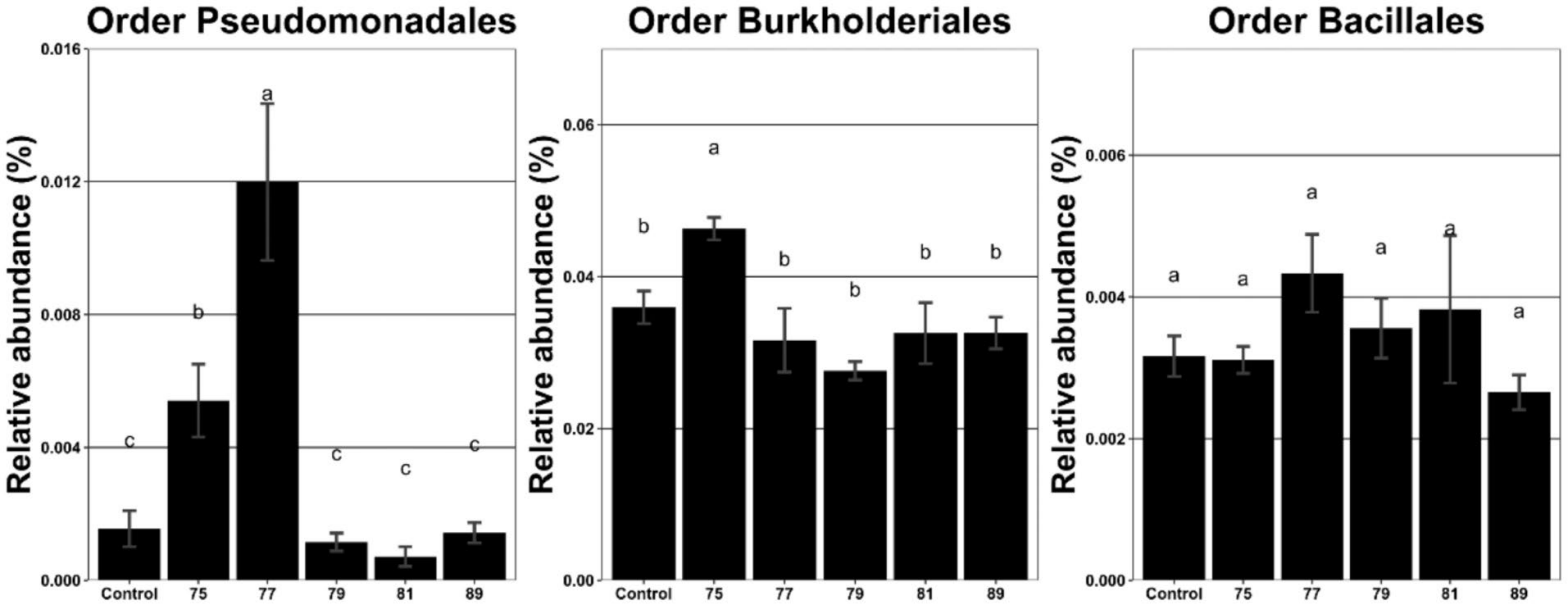
Relative abundance (%) of the bacterial orders Pseudomonadales, Burkholderiales, and Bacillales in the rhizosphere of wild-simulated ginseng after inoculation with different bacterial strains (*n* = 5). Different letters above the bars indicate significant differences based on Duncan’s multiple range test following ANOVA (*p* < 0.05).

The effects of continuous inoculation of each strain on soil microbial communities were assessed using metagenomic analysis. Inoculation with strains 75 and 77 significantly increased the relative abundance of Pseudomonadales compared with the control, the order that includes strains 75 and 77 (Figure 2). In contrast, inoculation with strains 79 and 89 (order Burkholderiales) and strain 81 (order Bacillales) did not significantly alter the abundance of their respective orders. Pearson correlation analysis between microbial orders and growth parameters revealed that the abundance of Pseudomonadales was positively correlated with root length, whereas that of Burkholderiales was positively correlated with dry weight (Table 1).

**Table 1:** Pearson correlation coefficients between the relative abundance of three bacterial orders (Pseudomonadales, Burkholderiales, and Bacillales) and various root growth parameters of wild-simulated ginseng.

Order		Root length (cm)	Root diameter (mm)	Root weight (g)	Number of root hair	Fresh weight (g)	Dry weight (g)
Pseudomonadales	Coefficient	**0.344** ^*^	0.065	0.166	−0.054	0.126	0.126
	*p* value	**0.014**	0.652	0.250	0.707	0.385	0.382
Burkholderiales	Coefficient	−0.028	0.091	0.239	0.051	0.245	**0.303** ^*^
	*p* value	0.846	0.529	0.094	0.724	0.087	**0.032**
Bacillales	Coefficient	−0.122	−0.035	−0.035	0.027	−0.067	−0.003
	*p* value	0.398	0.811	0.809	0.851	0.646	0.985

Asterisks (*) indicate statistically significant correlations (*p* < 0.05, *n* = 5). Coefficient values represent Pearson correlation coefficients; *p* values indicate the significance of each correlation. Bold values indicate significant differences.

**Figure 3:**
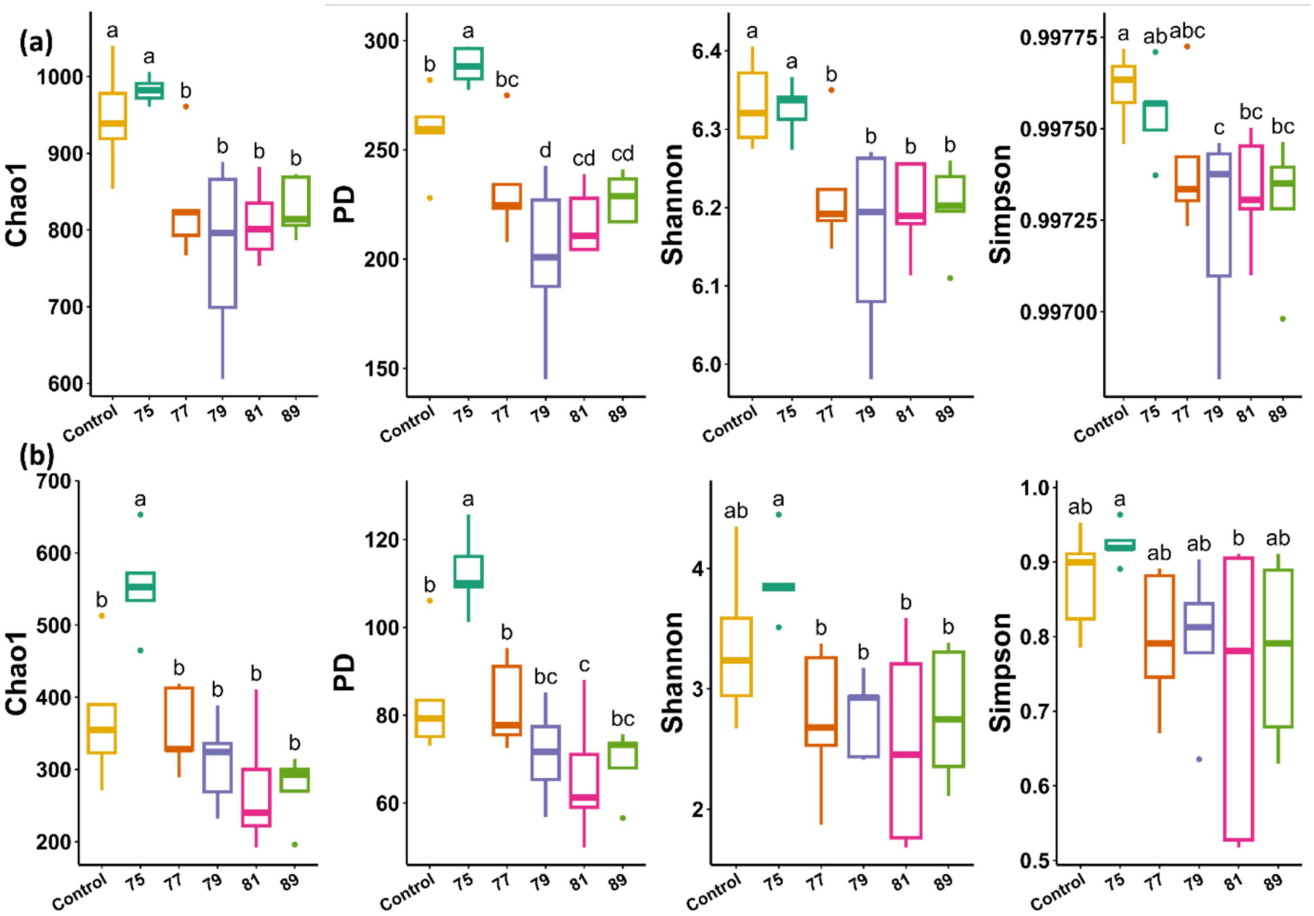
Alpha diversity indices of microbial communities in the rhizosphere soil of wild-simulated ginseng after inoculation with different bacterial strains (*n* = 5). **(a)** Bacterial alpha diversity based on Chao1, PD, Shannon, and Simpson indices. **(b)** Fungal alpha diversity based on the same indices. Different letters indicate significant differences according to Duncan’s multiple range test (*p* < 0.05).

**Figure 4:**
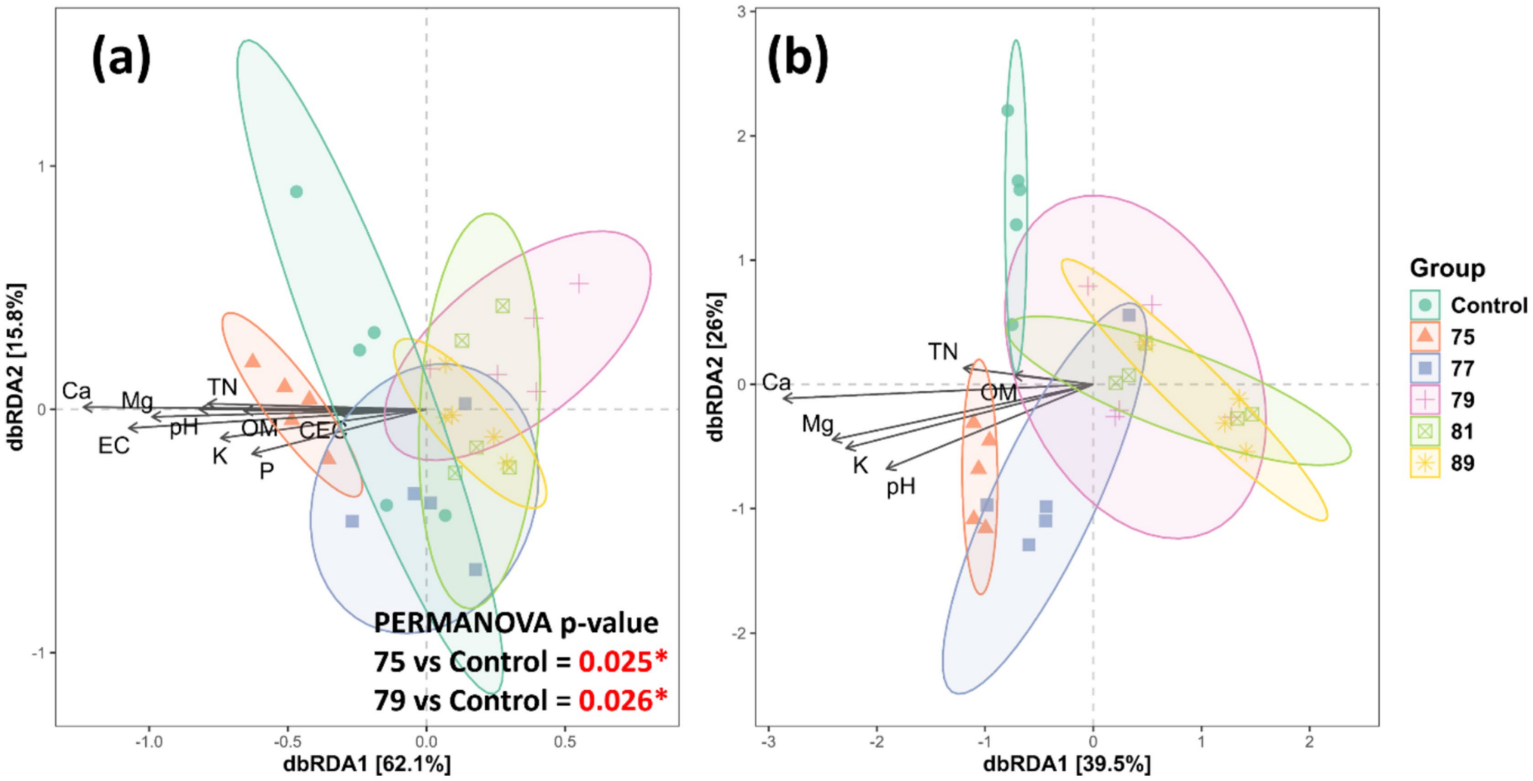
Beta diversity of microbial communities associated with the rhizosphere soil of wild-simulated ginseng across bacterial inoculation treatments (*n* = 5). dbRDA plots showing bacterial **(a)** and fungal **(b)** community structures constrained by soil chemical properties. Arrows indicate the direction and strength of the correlation between environmental variables and community composition. PERMANOVA results denote significant differences in microbial community compositions between strain 75 or 79 and the control (*p* < 0.05).

In the bacterial alpha diversity analysis, all treated plots, except for that treated with strain 75, exhibited significantly decreased alpha diversity across all indices compared with the control. In contrast, bacterial alpha diversity was maintained or slightly increased in the plot treated with strain 75 (Figure 3a). In the fungal alpha diversity analysis, only the plot treated with strain 75 showed significantly increased alpha diversity (Chao1 and PD indices) (Figure 3b). In the bacterial beta diversity analysis, only the plots treated with strains 75 and 79 differed significantly from the control plot. Mantel tests revealed that the bacterial community structure was significantly correlated with soil chemical factors including pH, EC, Mg, Ca, K, P, OM, TN, and CEC (Figure 4a). In contrast, in the fungal beta diversity analysis, none of the treated plots differed significantly from the control plot. The fungal community structure was significantly correlated with OM, TN, Ca, Mg, K, and pH (Figure 4b).

**Figure 5:**
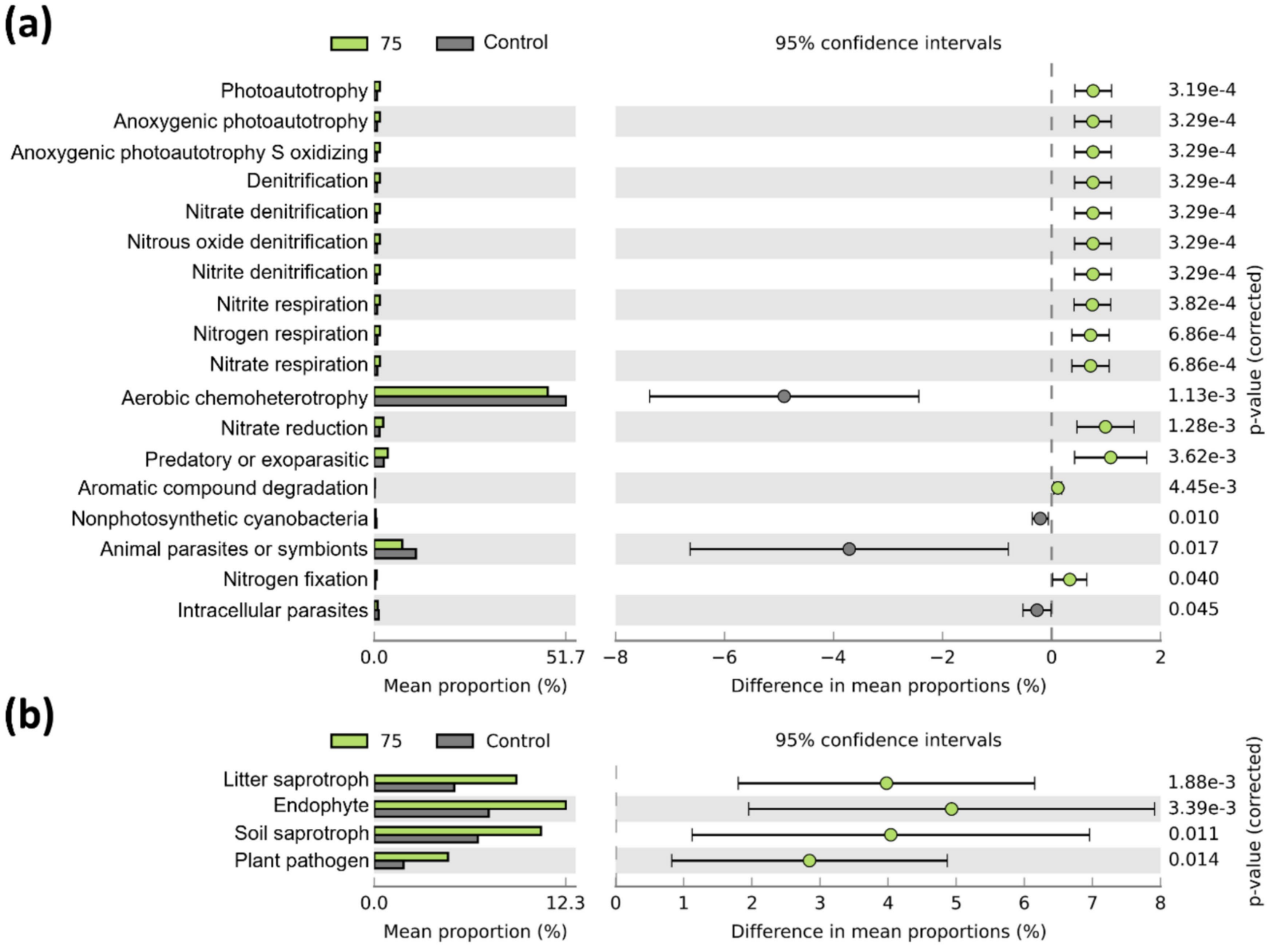
Predicted microbial functional traits based on sequencing data (*n* = 5). **(a)** Bacterial functions were predicted using FAPROTAX, and **(b)** fungal functions were predicted using FUNGuild. Only significantly different functional traits between strain 75 and the control (adjusted *p* < 0.05) are shown.

**Figure 6:**
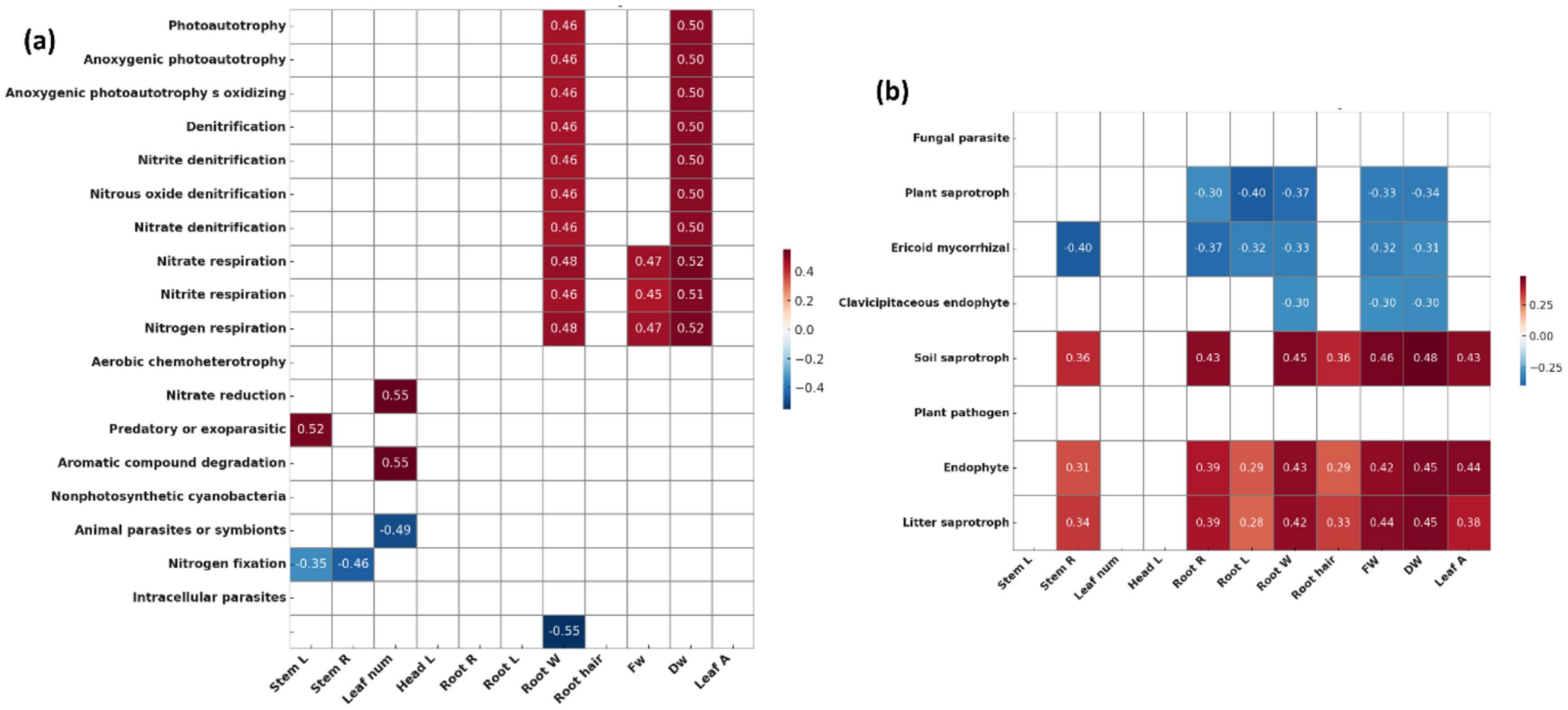
Correlation heatmaps between microbial functional traits and plant growth parameters (five replicates per treatment, *n* = 30 in total). **(a)** Bacterial functional traits predicted by FAPROTAX and **(b)** fungal functional traits predicted by FUNGuild were analyzed using Pearson correlation analysis. Color scale represents Pearson correlation coefficients (*p* values), with red indicating positive correlations and blue indicating negative correlations. Only statistically significant correlations (*p* < 0.05) are color-coded. Growth parameters on the x-axis include stem length (Stem_L), stem diameter (Stem_R), leaf number (Leaf_num), rhizome head length (Head_L), root length (Root_L), root diameter (Root_R), root weight (Root_W), number of root hair (Hair_num), fresh weight (FW), dry weight (DW), and leaf area (Leaf_A).

Bacterial functional traits related to nitrogen cycling and nitrogen fixation were enriched in the plot treated with strain 75 (Figure 5a). This plot also exhibited a higher relative abundance of fungi associated with OM decomposition (litter and soil saprotrophs) and endophytic fungi compared with the control (Figure 5b). However, several of the other treated plots showed decreased abundance of nitrogen-fixing bacteria (Supplementary Figure S4). Bacterial functional traits associated with nitrogen cycling and photoautotrophy were positively correlated with root weight and dry weight (Figure 6a). Fungal functional traits associated with soil saprotrophs, endophytes, and litter saprotrophs were positively correlated with plant growth parameters, whereas those associated with plant saprotrophs, ericoid mycorrhizae, and clavicipitaceous endophytes were negatively correlated with plant growth parameters (Figure 6b).

### 3.5 Network analysis of soil microbial community

In the bacterial network, a total of 118 nodes, 211 interactions, and nine modules were identified. In the fungal network, a total of 88 nodes, 185 interactions, and seven modules were identified (Supplementary Figure S6). Modularity analysis was performed to assess the effect of each node within and between modules. Subsequently, seven OTUs in the bacterial network and 14 OTUs in the fungal network were classified as keystone taxa (module hubs or connectors) (Supplementary Figure S7; Supplementary Tables S3, S4).

To determine the effect of inoculation of each strain on these keystone taxa, Pearson correlation analysis was performed between the taxonomic orders of the selected strains and those of the key taxa. In the bacterial community, OTU162 (order Elsterellales) and OTU400 (order Clostridiales) exhibited negative correlations with Pseudomonadales and Burkholderiales; OTU627 (order Bryobacterales) and OTU652 (order Acetobacterales) also exhibited negative correlations with Burkholderiales and Bacillales (Supplementary Figure S8a). OTU825 (order Entotheonellales) exhibited a positive correlation with Bacillales. Moreover, OTU1433 (order Rhizobiales) was positively correlated with all strain-associated orders, suggesting a potential central role in linking multiple taxa (Supplementary Figure S8a). In contrast, in the fungal community, no keystone taxa correlated with Pseudomonadales or Bacillales were identified. Instead, OTU3 (order Spizellomycetales), OTU63 (order Russulales), OTU103 (order Saccharomycetales), OTU136 (order Orbiliales), and OTU318 (order Pleosporales) were positively correlated with Burkholderiales, indicating selective associations between fungal taxa and this bacterial order (Supplementary Figure S8b).

These results indicate that inoculation with strain 75 and related Pseudomonadales may promote beneficial associations with key taxa such as Rhizobiales, which are known to enhance nitrogen fixation and plant productivity (Garrido-Oter et al., 2018). In addition, Burkholderiales-associated strains were linked with specific fungal key taxa, indicating selective interactions that may also contribute to nutrient cycling and soil ecosystem functioning (Zhang et al., 2018; Kisło et al., 2024).

### 3.6 Soil property analysis

As a result of soil property analysis, plots treated with strain 75 and 77 showed a similar level of organic matter (OM), potassium (K), total nitrogen (TN) and cation exchange capacity (CEC) to the untreated control, while phosphorus (P) were higher. In particular, P was highest in the plots treated with strains 75 and 77. Magnesium (Mg) and calcium (Ca) were significantly higher only in the strain 75 treatment. Soil pH and electrical conductivity (EC) were also higher in the strain 75 treatment relative to the other treatments. By contrast, the strain 79, 81, and 89 treatments exhibited lower soil fertility indices, including OM, P, TN, K, Ca, Mg, EC and CEC (Table 2).

**Table 2:** Soil physicochemical properties.

Strain	OM	TN	P	K	Ca	Mg	Na	CEC	pH	EC
75	9.15^a^ (1.24)	0.29^a^ (0.03)	66.66^a^ (9.28)	0.23^a^ (0.02)	5.93^a^ (1.56)	0.91^a^ (0.2)	0.05^c^ (0.01)	21.73^a^ (2.61)	4.46^a^ (0.12)	1.24^a^ (0.09)
77	9.59^a^ (1.55)	0.3^a^ (0.04)	65.71^a^ (7.14)	0.19^a^ (0.05)	3.54^b^ (1.56)	0.71^ab^ (0.23)	0.07^ab^ (0.01)	21.49^a^ (2.46)	4.16^b^ (0.12)	1.16^ab^ (0.13)
79	6.16^b^ (2.11)	0.22^b^ (0.05)	36.95^c^ (8.52)	0.14^b^ (0.02)	1.78^c^ (0.95)	0.41^c^ (0.12)	0.04^c^ (0.01)	17.26^b^ (3.13)	4.06^b^ (0.07)	0.86^d^ (0.16)
81	5.97^b^ (0.31)	0.21^b^ (0.01)	44.85^bc^ (7.86)	0.15^b^ (0.03)	1.89^c^ (0.86)	0.46^c^ (0.12)	0.09^ab^ (0.1)	17.35^b^ (1.08)	4.09^b^ (0.13)	0.86^d^ (0.03)
89	6.92^b^ (1.78)	0.23^b^ (0.04)	47.43^bc^ (7.95)	0.15^b^ (0.01)	1.57^c^ (0.37)	0.42^c^ (0.07)	0.05^c^ (0.01)	18.06^b^ (2.51)	4.07^b^ (0.04)	0.93^cd^ (0.09)
Con	9.39^a^ (1.97)	0.3^a^ (0.06)	50.46^b^ (4.39)	0.19^a^ (0.03)	4.29^b^ (1.04)	0.68^b^ (0.16)	0.14^a^ (0.11)	21.4^a^ (2.21)	4.17^b^ (0.03)	1.03^bc^ (0.1)

Value in parentheses is standard error (*n* = 5). Different letters indicate a significant difference at *p* < 0.05 based on the results of ANOVA using Duncan’s studentized range test. OM, Organic matter; TN, Total nitrogen; P, Phosphorus; K, Potassium; Ca, Calcium; Mg, Magnesium; Na, Sodium; CEC, Cation exchange capacity; EC, Electrical conductivity.

## 4 Discussion

**Figure 7:**
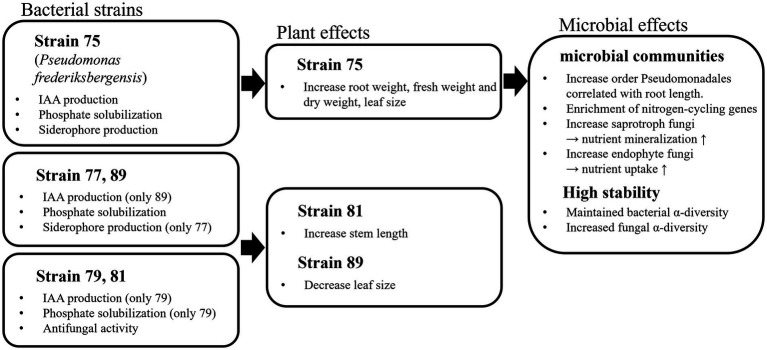
Mechanistic diagram showing how selected rhizosphere bacteria influence wild-simulated ginseng growth through plant growth promotion and soil microbial community shifts.

In this study, various strains were isolated and inoculated into field-grown wild-simulated ginseng to evaluate their effects. In the field tests, strain 75 was the most effective in promoting the initial growth of wild-simulated ginseng and demonstrated high applicability. Metagenomic analysis revealed that inoculation with strain 75 induced shifts in the soil microbiome, such as increasing the abundance of Pseudomonadales and enhancing community stability by maintaining or increasing alpha diversity. Moreover, strain 75 improved the soil environment by enhancing nitrogen fixation and nutrient cycling (Figure 7).

Strain 75 was identified as *P. frederiksbergensis*, which belongs to the order Pseudomonadales. In a previous study, Pseudomonadales species were found to be more abundant in the rhizosphere than in bulk soil. Moreover, a meta-analysis highlighted that Pseudomonadales, together with Bacillales, are important order associated with PGPR (Zhao et al., 2023). Pseudomonadales species also produce phytohormones, solubilize phosphate, fix nitrogen, and enhance tolerance to abiotic stress (Zheng et al., 2024). Therefore, numerous studies have been conducted using Pseudomonadales species to improve crop yield and health (Garcia-Villaraco et al., 2024; Ramírez et al., 2024). Among these, *P. frederiksbergensis* has also been reported to be a member of the PGPR family (Zeng et al., 2016; Chatterjee et al., 2017). Inoculation with strain 75 altered bacterial community composition relative to that of the control plot and was correlated with changes in soil chemical properties. Thus, the continuous application of strain 75 could beneficially shift the soil microbiome toward a plant growth-promoting state.

Photoautotrophic bacteria synthesize OM from inorganic compounds using light. These functions facilitate biomass cycling in soil and support the role of these bacteria as biofertilizers through the synthesis of carotenoids in photobioreactors (Scognamiglio et al., 2021). Denitrification activity increases as bacterial metabolism intensifies during vigorous plant growth (Robinson and Conroy, 1998). In addition, compounds and enzymes required for plant–microbe interactions are produced during this process, thereby improving the rhizosphere environment (Hamada and Soliman, 2023). Nitrate, nitrite, and nitrogen respiration are processes in the nitrogen cycle that regulate nitrogen uptake by altering nitrogen forms. Nitrogen metabolism also generates signaling compounds essential for plant–microbe interactions, such as nitric oxide, which promotes plant growth and enhances resistance (Kraft et al., 2014; Yuan et al., 2020; Pande et al., 2021). The order Burkholderiales, associated with these functions, has also been reported to include important PGPR (Zhao et al., 2023).

Soil, leaf litter, and plant saprotrophic fungi make nutrients available to plants through the decomposition of OM (Lebreton et al., 2021). However, plant saprotrophic fungi are negatively correlated with plant growth, likely due to an increased abundance of tissue-decomposing fungi following pathogen attack or abiotic stress (Tao et al., 2025). Ericoid mycorrhizal fungi and clavicipitaceous endophytic fungi form symbiotic associations with members of the families Ericaceae and Poaceae, respectively. In general, endophytic fungi interact with plants to facilitate nutrient uptake or promote growth (Baldrian, 2017). Nonetheless, fungi exhibiting strict host specificity may behave as toxins or pathogens in non-host plants (Trognitz et al., 2016).

In microbial communities, a module is defined as a group of microbial taxa that interact strongly with each other. Such modules are thought to perform similar functions in the environment (Banerjee et al., 2018). Taxa that are crucial for regulating the functions of a module or maintaining its stability are called module hubs, whereas those that play a key role in facilitating interactions between modules are called connectors. Taxa that fulfill both roles are known as network hubs. Collectively, these groups are referred to as keystone taxa. Studies have revealed that changes in these keystone taxa can explain community shifts more effectively than analyses involving the entire taxonomic assemblage (Herren and McMahon, 2017). Moreover, the loss of keystone taxa can induce significant alterations in microbial community composition and functional traits (Banerjee et al., 2018).

In this study, inoculation with strain 75 increased the abundance of Pseudomonadales, which corresponded with an increase in OTU1433 (order Rhizobiales) and decreases in OTU162 (order Elsterales) and OTU400 (order Clostridiales). The order Elsterales has been previously reported to be negatively correlated with nutrient elements and to exhibit opposite effects on maize growth (Hong et al., 2021; Wang et al., 2022). Moreover, members of the order Rhizobiales actively interact with plants and facilitate soil nitrogen fixation, thereby enhancing plant productivity (Jones, 2015). Therefore, the observed changes in these keystone taxa are considered to positively impact the growth of wild-simulated ginseng. In contrast, members of the order Clostridiales are involved in the hydrolysis of cellulose and lignocellulose (Sun et al., 2016). Therefore, it is necessary to monitor changes in nutrient cycling following inoculation with strain 75. In the fungal community, the increase in Pseudomonadales exhibited minimal correlations with keystone taxa, indicating that inoculation with strain 75 poses little risk of disrupting the fungal community.

## 5 Conclusion

This study demonstrated the potential of rhizosphere bacteria as plant growth-promoting rhizobacteria (PGPR) in wild-simulated ginseng cultivation. Among the strains tested, strain 75 (*Pseudomonas frederiksbergensis*) consistently enhanced root biomass and maintained soil microbial diversity, highlighting its novelty and comparative effectiveness relative to other strains such as 81 and 89. These findings highlight the significance of PGPR application for sustainable ginseng cultivation, as strain 75 promoted plant growth through hormone production, nutrient solubilization, and stabilization of microbial communities. Such effects suggest that PGPR can be developed as biocontrol agents specifically for wild-simulated ginseng, improving root biomass and quality while supporting the sustainable management of this medicinal crop. Moreover, the observed effects on nutrient cycling and microbial diversity indicate additional environmental benefits, such as reduced reliance on chemical fertilizers and improved soil fertility.

Nevertheless, this study was limited to pot-scale experiments under specific soil conditions, and longer-term field validation is required. Future research should evaluate the performance of these strains in diverse environments and investigate their molecular mechanisms to optimize their application across crops.

## Data Availability

The bacterial and fungal metagenomic datasets supporting this study have been submitted to the NCBI SRA under BioProject accession numbers PRJNA1357602 and PRJNA1357594.
