# Supplementary material for: Isolation of plant growth-promoting rhizobacteria from wild-simulated ginseng and evaluation of soil health following its application in the field
Source: Front Microbiol. 2025 Nov 25;16:1682016. doi: 10.3389/fmicb.2025.1682016 (PMC12685894; doi:10.3389/fmicb.2025.1682016)
Supplement: Supplementary file 1 [file Data_Sheet_1.docx]

**Supplementary Table 1.** Summary of enzymatic activities, phytohormone production, and antifungal activity of each bacterial strain.

| **Strain** | **IAA**  **(ppm)** | **Phosphate**  **solubilizing** | **Siderophore** | **Protease** | **Cellulase** | **Chitinase** | **Nitrogen**  **fixation** | **Fungal**  **antagonism** |
| --- | --- | --- | --- | --- | --- | --- | --- | --- |
| 73 | - | + | - | + | - | + | - | - |
| 75 | 19.7±13.9 | + | + | + | - | - | - | - |
| 77 | - | + | + | + | - | - | - | - |
| 79 | 7.4±1.9 | + | - |  | - | - | + | + |
| 80 | - | - | - | + | - | - | - | - |
| 81 | - | - | - | + | + | - | - | + |
| 82 | - | + | + | + | - | - | - | - |
| 83 | - | + | + | + | - | - | - | - |
| 85 | - | + | - | + | + | - | - | + |
| 87 | - | - | - | + | - | - | - | - |
| 89 | 19.2±4.2 | + | - | - | - | - | - | - |
| 93 | - | -- | - | - | - | - | - | - |
| 94 | - | + | - | - | - | - | - | - |
| 95 | - |  | - | - | - | - | - | - |
| 96 | - | + | + | - | - | - | - | - |
| 99 | - | - | - | + | - | - | - | - |
| 200 | - | - | - | + | + | - | - | + |
| 201 | - | - | - | + | - | - | - | - |
| 205 | - | - | - | - | - | - | - | - |
| 210 | 6.9±2 | - | - | + | + | - | - | + |


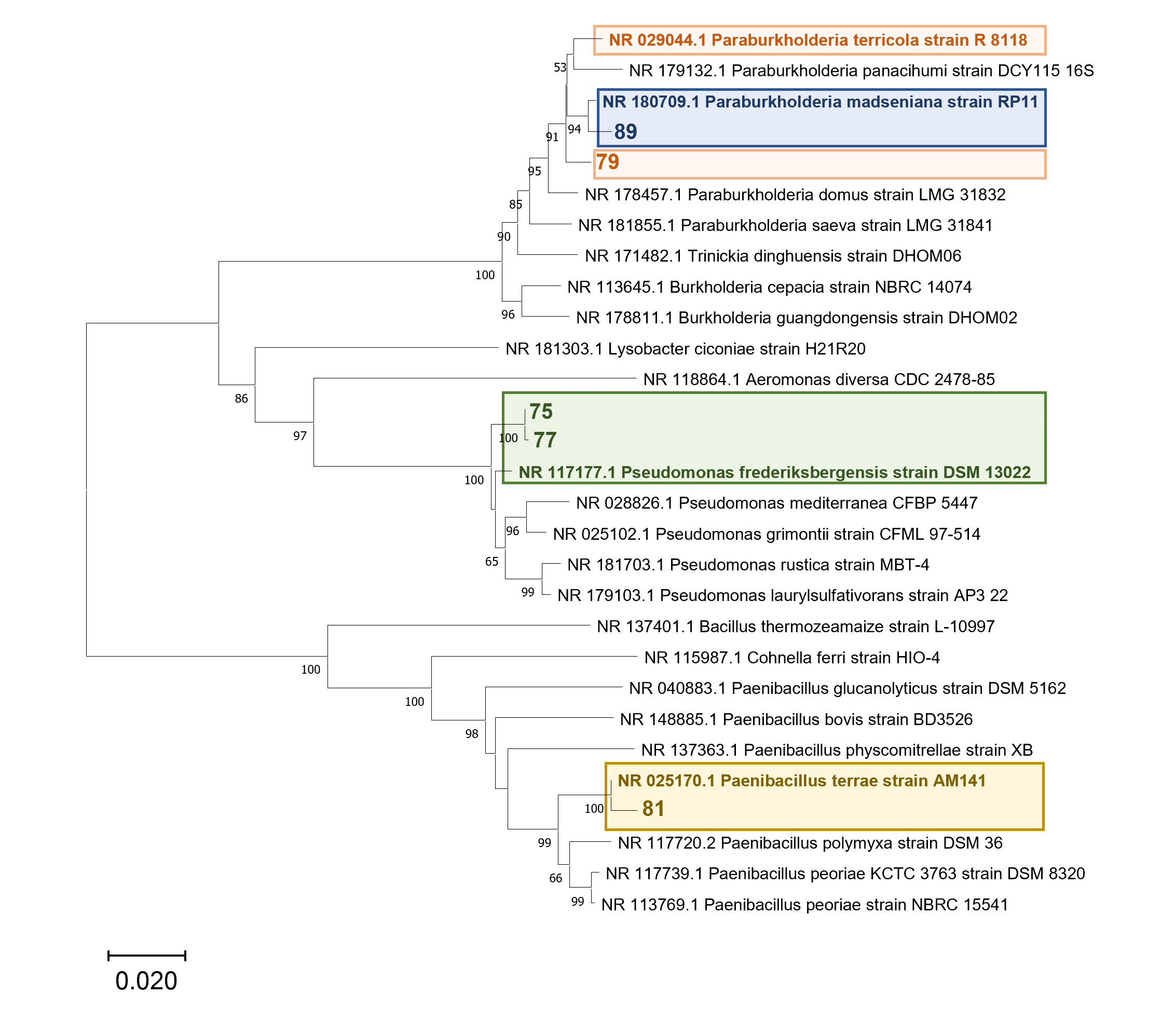


**Supplementary Figure 1.** Phylogenetic tree of bacterial strains isolated in this study. The tree is based on 16S rRNA gene sequences. Strains isolated in this study and their closest NCBI-registered strains are shown in bold.


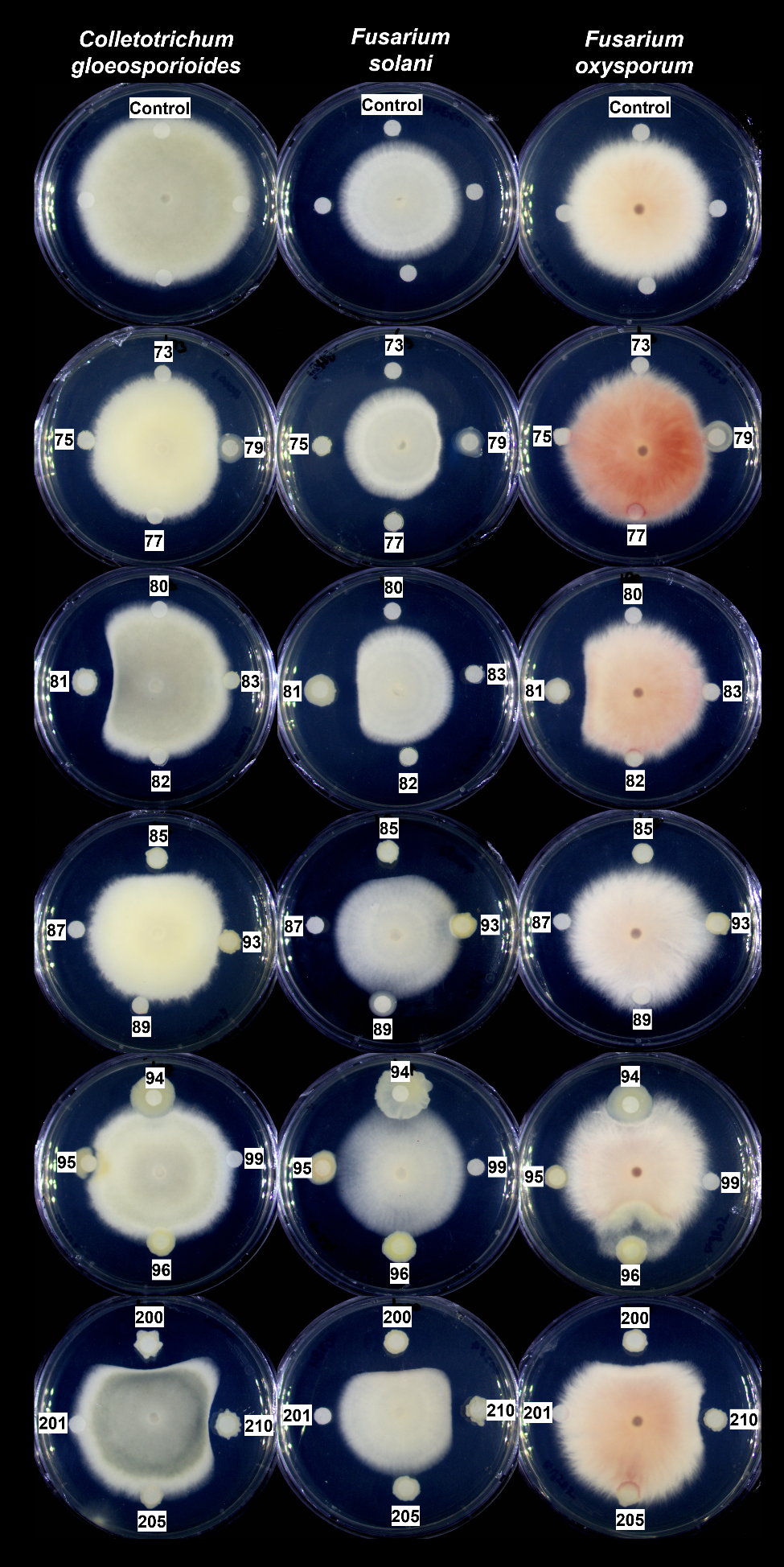


**Supplementary Figure 2.** Antagonistic activity of bacterial strains against phytopathogenic fungi.


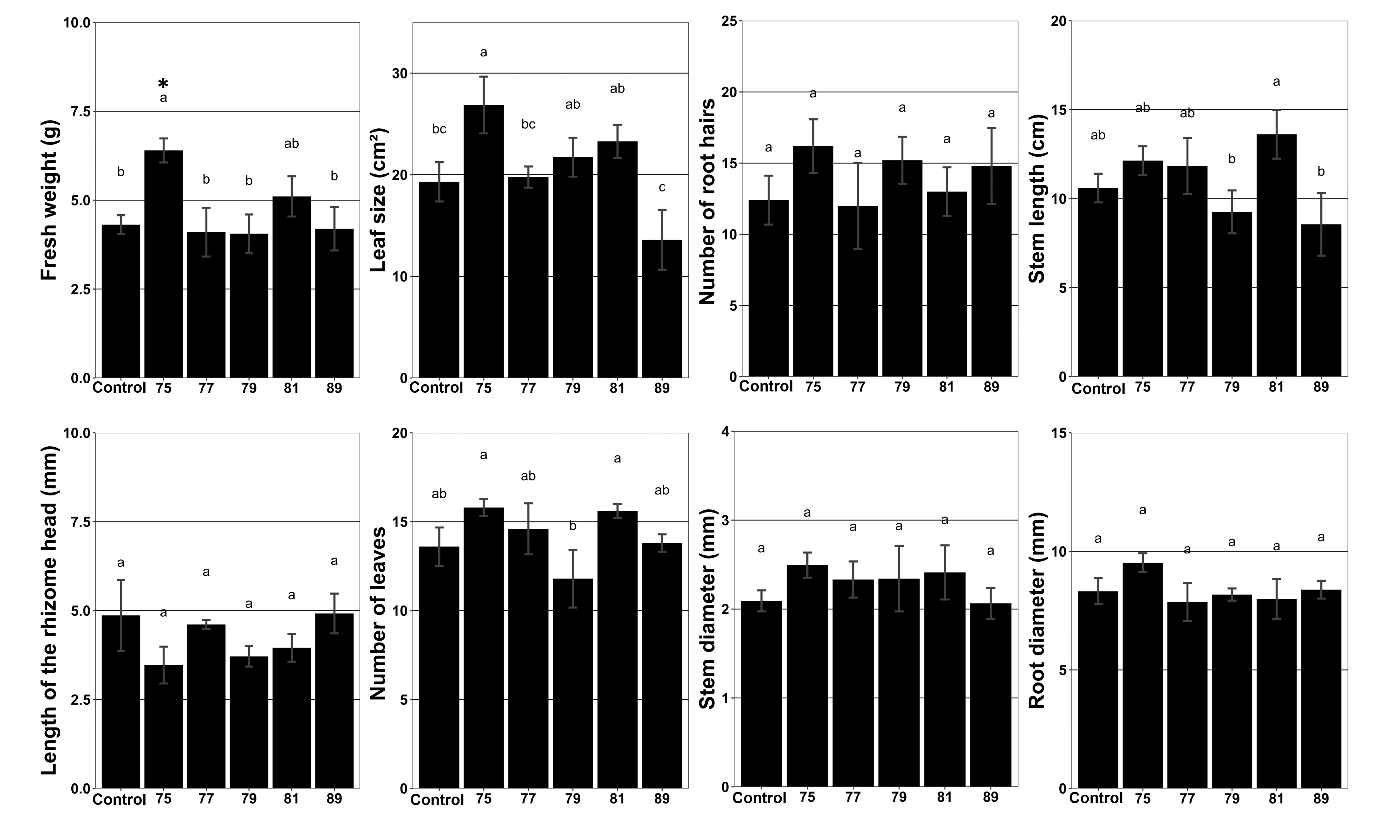


**Supplementary Figure 3.** Aboveground growth characteristics of mountain ginseng in response to inoculation with different bacterial strains. Different letters above the bars indicate significant differences based on Duncan’s multiple range test following ANOVA (p < 0.05). Asterisks (*) indicate significant differences compared to the control group based on t-test (p < 0.05).

**Supplementary Table 2.** Pearson correlation coefficients between the relative abundance of three bacterial orders (Pseudomonadales, Burkholderiales, and Bacillales) and various aboveground growth parameters of mountain ginseng. Asterisks (*) indicate statistically significant correlations (p < 0.05).

| **Order** |  | **Stem**  **length**  **(cm)** | **Stem**  **diameter**  **(mm)** | **Number**  **of**  **leaves** | **Leaf**  **area**  **(cm2)** | **Rhizome**  **head**  **length (cm)** |
| --- | --- | --- | --- | --- | --- | --- |
| Pseudomonadales | Coefficient | **0.215** | 0.117 | 0.156 | 0.131 | 0.046 |
|  | *p*-value | **0.134** | 0.419 | 0.279 | 0.365 | 0.753 |
| Burkholderiales | Coefficient | 0.053 | 0.029 | 0.112 | -0.003 | -0.162 |
|  | *p*-value | 0.713 | 0.841 | 0.439 | 0.982 | 0.261 |
| Bacillales | Coefficient | -0.062 | -0.136 | -0.107 | 0.007 | -0.217 |
|  | *p*-value | 0.671 | 0.347 | 0.460 | 0.959 | 0.129 |


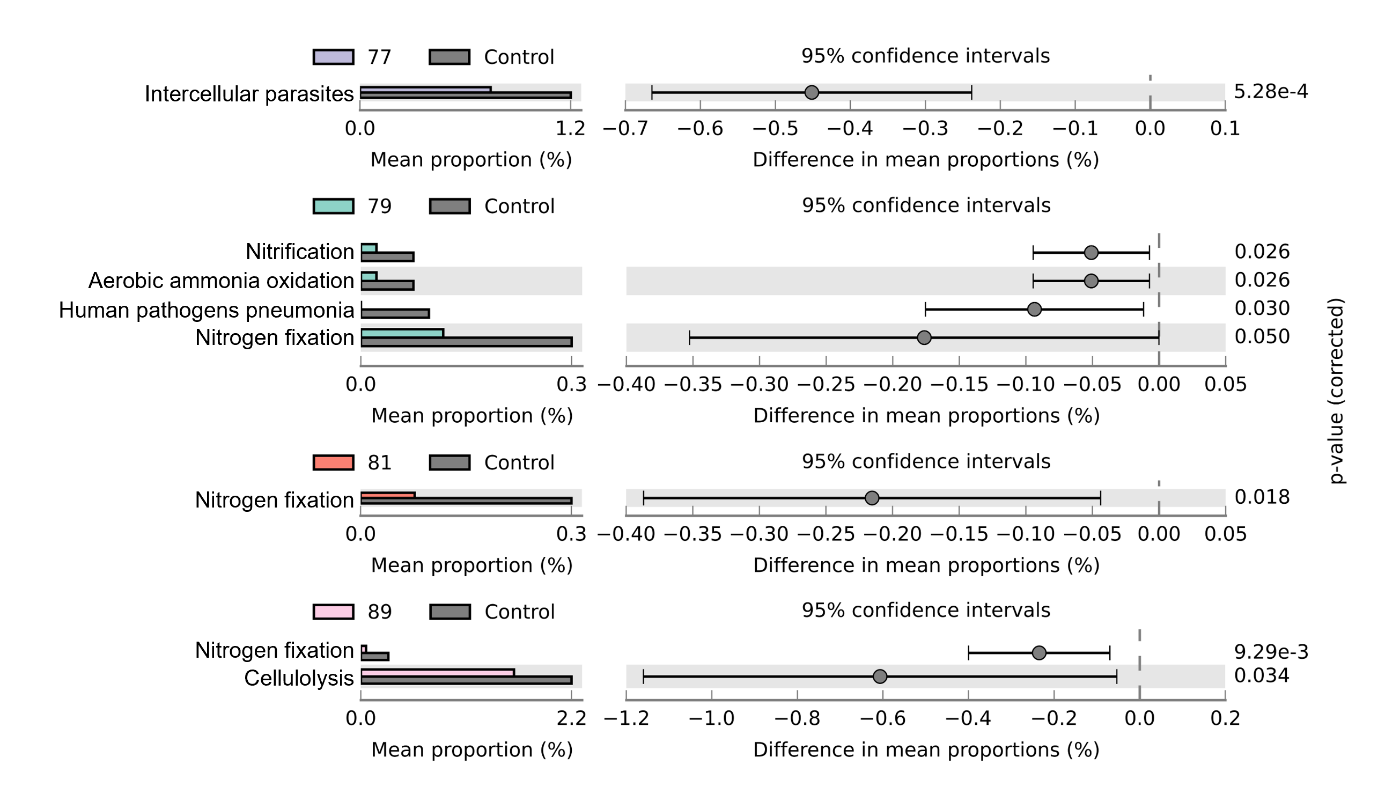


**Supplementary Figure 4.** Predicted functional traits of bacterial communities based on FAPROTAX analysis across different strains (77, 79, 81, 89, 200, and 210) compared to control. Only significantly different functions (p < 0.05) are shown.


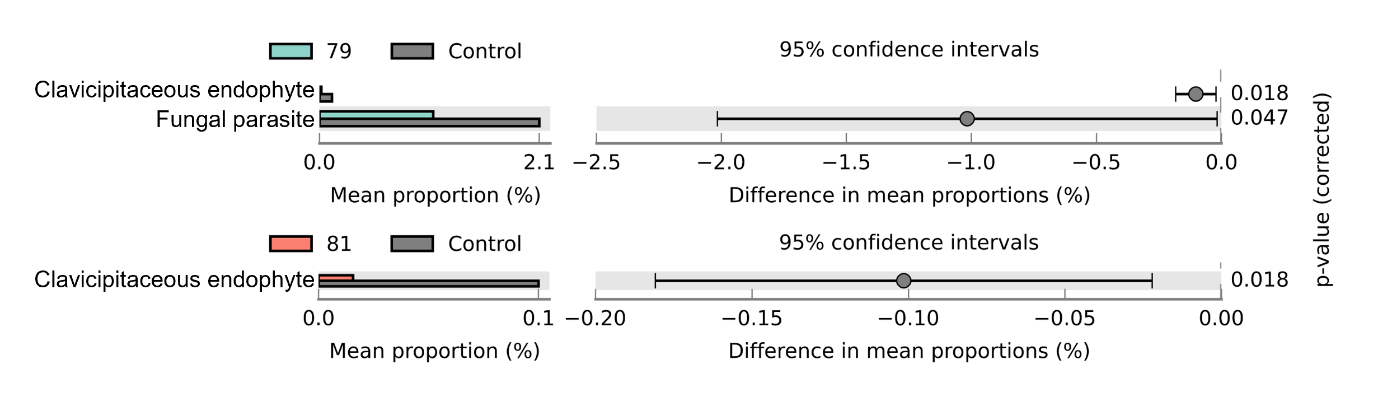


**Supplementary Figure 5.** Predicted fungal functional groups based on FUNGuild analysis across different strains (79, 81, 200, and 210) compared to the control. Only significantly different functions (p < 0.05) are shown.


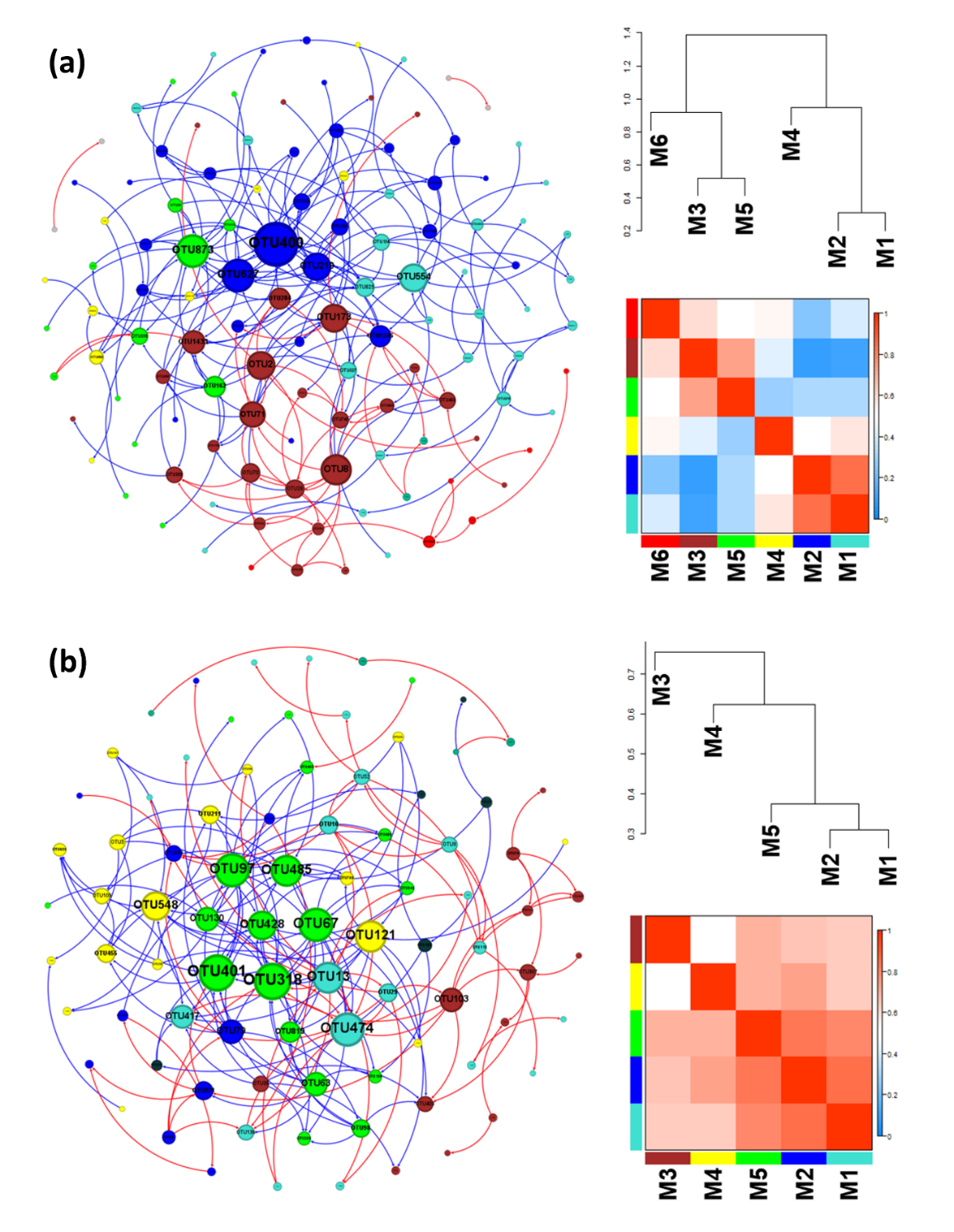


**Supplementary Figure 6.** Network analysis of microbial communities based on co-occurrence patterns. (a) Bacterial and (b) fungal community networks. Each node represents an OTU, and edges indicate significant correlations between OTUs: blue lines represent positive interactions, and red lines represent negative interactions. The size of each node reflects the number of interactions (degree). The right panels show module-based clustering dendrograms (top) and Pearson correlation heatmaps (bottom), representing the similarity among modules.


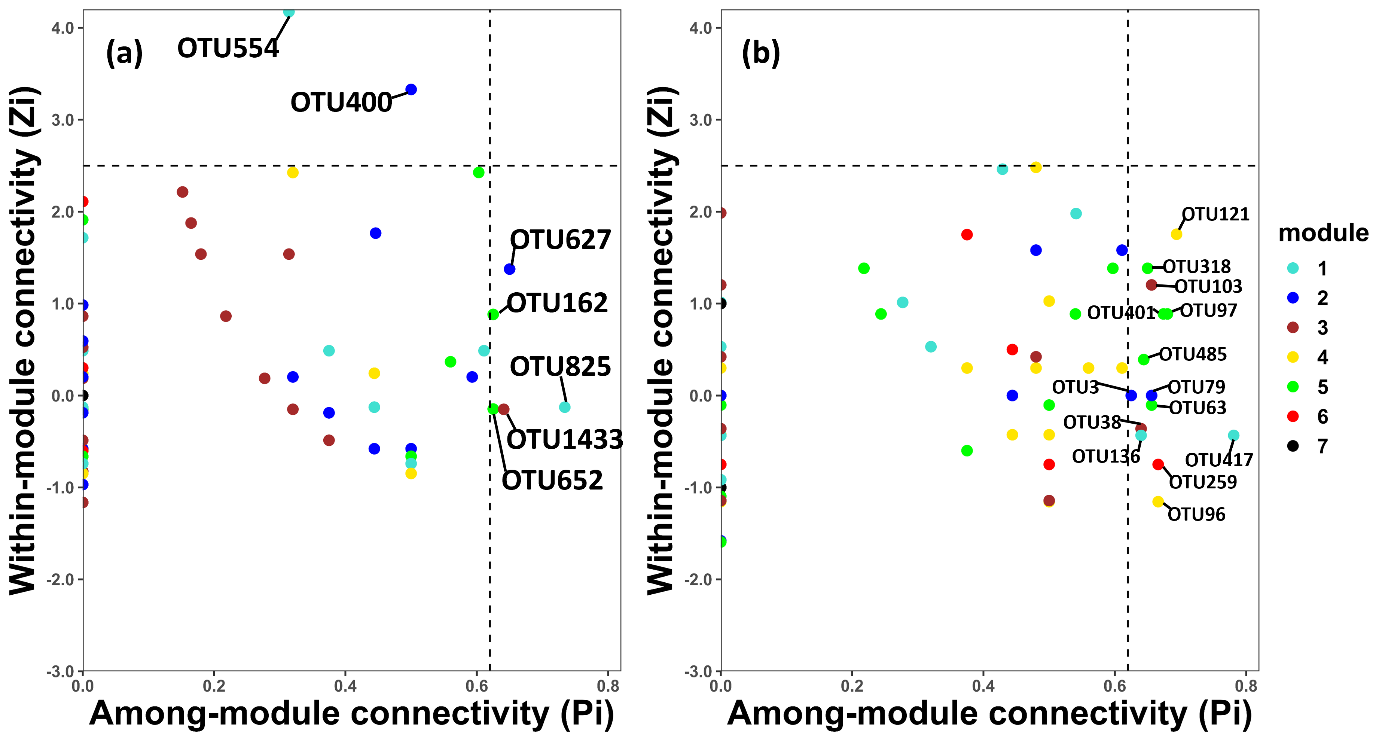


**Supplementary Figure 7.** Zi–Pi plots used to identify potential keystone taxa within microbial networks. (a) Bacterial and (b) fungal communities. Each point represents an OTU. OTUs were categorized into four topological roles based on their within-module connectivity (Zi) and among-module connectivity (Pi): (1) Peripherals (Zi < 2.5, Pi < 0.62), (2) Module hubs (Zi > 2.5, Pi < 0.62), (3) Connectors (Zi < 2.5, Pi > 0.62), and (4) Network hubs (Zi > 2.5, Pi > 0.62). Labeled OTUs represent those identified as connectors or module hubs.

| **ID** | **Module** | **class** | **order** | **family** | **genus** |
| --- | --- | --- | --- | --- | --- |
| OTU162 | 4 | Alphaproteobacteria | Elsterales | URHD0088 | URHD0088 |
| OTU400 | 1 | Clostridia | Clostridiales | Clostridiaceae | Clostridium_sensu |
| OTU554 | 0 | Planctomycetes | Isosphaerales | Isosphaeraceae | uncultured |
| OTU627 | 1 | Acidobacteriae | Bryobacterales | Bryobacteraceae | Bryobacter |
| OTU652 | 4 | Alphaproteobacteria | Acetobacterales | Acetobacteraceae | Acidocella |
| OTU825 | 0 | Entotheonellia | Entotheonellales | Entotheonellaceae | Entotheonellaceae |
| OTU1433 | 2 | Alphaproteobacteria | Rhizobiales | Methyloligellaceae | Methyloligellaceae |

**Supplementary Table 3.** Taxonomy information of each bacterial keystone taxon.

**Supplementary Table 4.** Taxonomy information of each fungal keystone taxon.

| **ID** | **Module** | **class** | **order** | **family** | **genus** |
| --- | --- | --- | --- | --- | --- |
| OTU3 | 2 | Spizellomycetes | Spizellomycetales | unidentified | unidentified |
| OTU38 | 3 | Lecanoromycetes | GS36 | unidentified | unidentified |
| OTU63 | 5 | Agaricomycetes | Russulales | Russulaceae | Russula |
| OTU79 | 2 | Dothideomycetes | Pleosporales | Didymellaceae |  |
| OTU96 | 4 | Sordariomycetes | Xylariales | Xylariaceae | unidentified |
| OTU97 | 5 | Agaricomycetes | Russulales | Russulaceae | Russula |
| OTU103 | 3 | Saccharomycetes | Saccharomycetales | unidentified | unidentified |
| OTU121 | 4 | Kickxellomycetes | Kickxellales | Kickxellaceae | Ramicandelaber |
| OTU136 | 1 | Orbiliomycetes | Orbiliales | unidentified | unidentified |
| OTU259 | 6 | Dothideomycetes | Venturiales | Venturiaceae | Sympodiella |
| OTU318 | 5 | Dothideomycetes | Pleosporales | Leptosphaeriaceae | Plenodomus |
| OTU401 | 5 | Pezizomycetes | Pezizales | Pezizaceae | unidentified |
| OTU417 | 1 | Agaricomycetes | Boletales | Boletaceae | unidentified |
| OTU485 | 5 | Agaricomycetes | Hymenochaetales | Hymenochaetaceae | Coltricia |


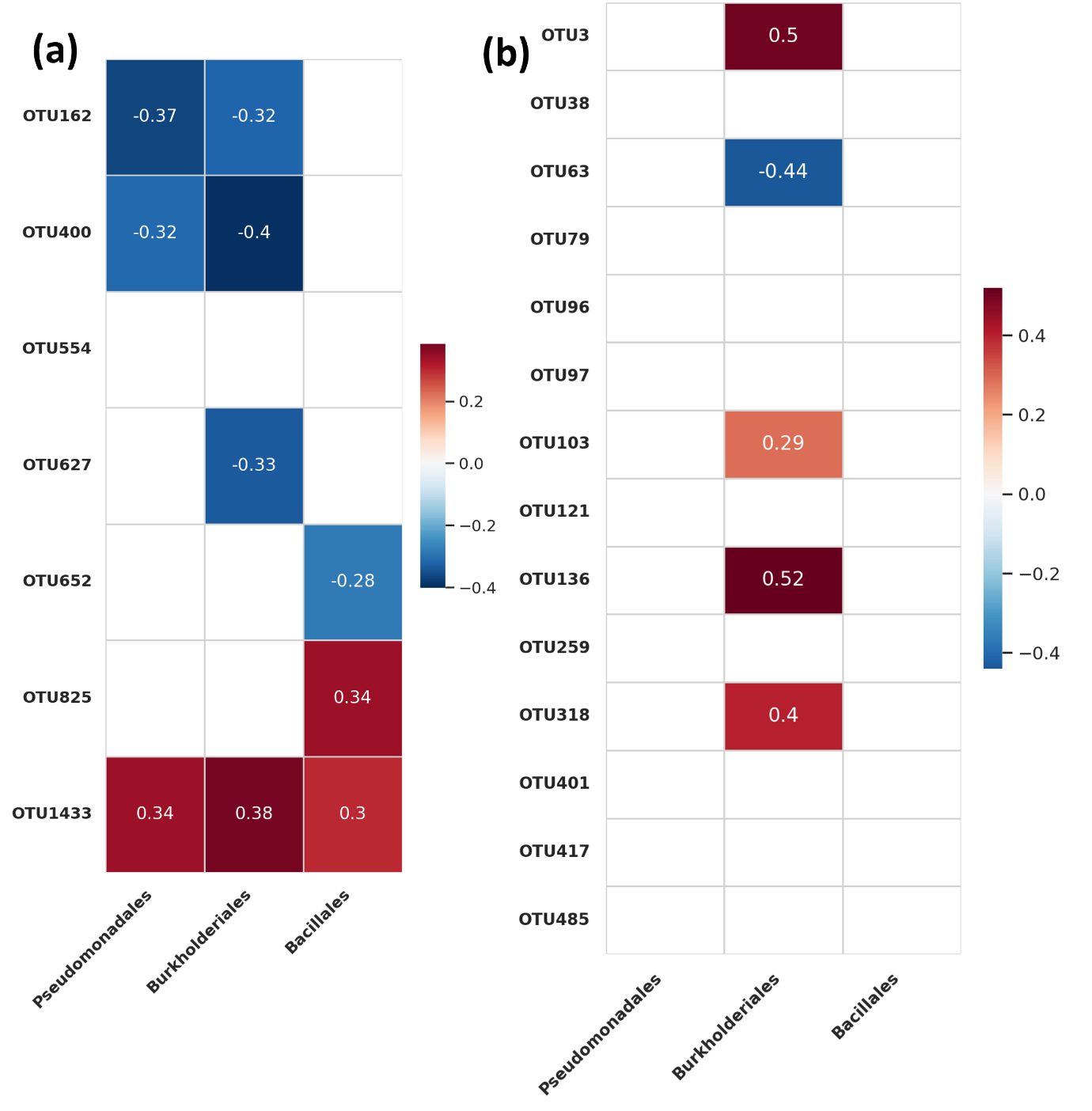


**Supplementary Figure 8.** Pearson correlation heatmaps between keystone taxa and each order of strain. (a) Keystone bacterial OTUs and their correlations with Pseudomonadales, Burkholderiales, and Bacillales. (b) Keystone fungal OTUs and their correlations with Pseudomonadales, Burkholderiales, and Bacillales.
